# Toll-Like Receptors in Natural Killer Cells and Their Application for Immunotherapy

**DOI:** 10.1155/2020/2045860

**Published:** 2020-01-04

**Authors:** Ji-Yoon Noh, Suk Ran Yoon, Tae-Don Kim, Inpyo Choi, Haiyoung Jung

**Affiliations:** ^1^Immunotherapy Research Center, Korea Research Institute of Bioscience and Biotechnology (KRIBB), 125 Gwahak-ro, Yuseong-gu, Daejeon 34141, Republic of Korea; ^2^Department of Functional Genomics, University of Science and Technology (UST), 113 Gwahak-ro, Yuseong-gu, Daejeon 34113, Republic of Korea

## Abstract

Innate immunity represents the first barrier for host defense against microbial infection. Toll-like receptors (TLRs) are the most well-defined PRRs with respect to PAMP recognition and induction of innate immune responses. They recognize pathogen-associated molecular patterns (PAMPs) and trigger innate immune responses by inducing inflammatory cytokines, chemokines, antigen-presenting molecules, and costimulatory molecules. TLRs are expressed either on the cell surface or within endosomes of innate immune cells. NK cells are one of the innate immune cells and also express TLRs to recognize or respond to PAMPs. TLRs in NK cells induce the innate immune responses against bacterial and viral infections via inducing NK cytotoxicity and cytokine production. In this review, we will discuss the expression and cellular function of TLRs in NK cells and also introduce some therapeutic applications of TLR agonists for NK cell-mediated immunotherapy.

## 1. Introduction

Cells involved in the innate immune response were initially speculated to nonspecifically eliminate microbes without presensitization; however, studies have reported that innate immune cells recognize microbial-associated or pathogen-associated molecular patterns (PAMPs) through their pattern recognition receptors (PRRs) including Toll-like receptors (TLRs), NOD-like receptors (NLRs), C-type lectin receptors (CLRs), and RIG-I-like receptors (RLRs) [1–3]. In particular, the discovery of TLRs in the mid-1990s indicated that pathogen recognition by the innate immune system actually depended on PRRs [1]. TLRs are the most well-defined PRRs with respect to PAMP recognition and induction of innate immune responses. TLRs are expressed either on the cell surface or within endosomes [4–6]. The interaction of different PAMPs with their cognate TLRs induces numerous intracellular signal transduction resulting in the activation of innate immune-related genes including those encoding inflammatory cytokines, costimulatory molecules, adhesion molecules, and antimicrobial mediators [6].

Innate immunity is coordinated by epithelial barriers, plasma proteins, and tissue-resident or circulating leukocytes including macrophages and neutrophils, dendritic cells (DCs), natural killer (NK) cells, and innate lymphoid cells [7]. Cells involved in the innate immune response recognize and prevent potential pathogen invasion that could result in infectious diseases [8, 9]. During an infection, cells involved in the innate immune response rapidly recognize and activate complex responses by recognizing such pathogens. Among these, NK cells are lymphocytes that mediate multiple effector functions and detect and eliminate transformed or virus-infected cells. However, NK cells reportedly express cell surface TLRs and directly recognize or respond to pathogens [6, 10]. TLR expression and function in NK cells were revealed owing to their potential involvement in the innate immune response to bacterial and viral infections via induction of NK cell-mediated cytotoxicity and cytokine production [6, 8, 11, 12].

Recent studies have furthered the current understanding of TLR expression and their critical role in NK cell-mediated innate immune responses against infections. This review is focused on recent advancements in studies on the expression and cellular function of TLRs in NK cell-induced antiviral and antibacterial responses. Furthermore, the potential applications of TLR agonists as potential boosters in stimulating immunological effector function of NK cells for cancer immunotherapy and infectious disease therapy are discussed herein.

## 2. General Features of TLRs and Their Ligands/Agonists

TLRs recognize conserved PAMPs, which serve as TLR agonists/ligands (TLRLs) [13, 14]. Some recent studies reported that endogenous, host-derived components, including fibrinogen, heat shock proteins, RNA, and DNA, also serve as TLRLs [14–16]. TLRs are expressed on cells involved in the innate immune response (myeloid and NK cells) and some cells of the adaptive immune system (regulatory and activated T cells) and mediate innate immune responses against microbial pathogens and induce adaptive immune responses [16, 17].

**Figure 1:**
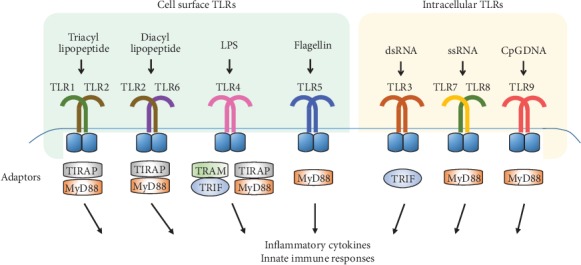
PAMP recognition by TLRs and adaptor proteins to mediate cellular signaling pathways. TLR members can be divided into cell surface types (TLR1, 2, 4, 5, and 6) and endosome types (TLR3, 7, 8, and 9). TLRs form homo- or heterodimer and have their respective ligands to be activated. After ligand binding to TLRs, TLRs dimerize and undergo the conformational change to recruit downstream adaptor proteins including myeloid differentiation primary response gene 88 (MyD88), TIR domain-containing adaptor protein (TIRAP)/MyD88-adaptor-like (Mal), TIR domain-containing adaptor inducing IFN-*β* (TRIF)/TIR domain-containing adaptor molecule-1 (TICAM-1), and TRIF-related adaptor molecule (TRAM).

Ten and 13 TLRs have been identified in humans and mice, respectively, with TLR1–TLR9 being conserved in both species. Mouse TLR10 is not functional owing to retrovirus insertion, and TLR11, TLR12, and TLR13 have been lost from the human genome [1, 18]. TLRs are type I transmembrane proteins with ectodomains containing leucine-rich repeats (LRR) that mediate PAMP recognition, transmembrane domains, and a conserved region of ~200 aa intracellular Toll-interleukin 1 (IL-1) receptor (TIR) domains required for downstream signal transduction [1, 13, 19]. All TLRs induce the myeloid differentiation primary response protein 88- (MyD88-) dependent pathways except TLR3 [20]. These sensors, TLRs, are differentially expressed among immune cells and have distinct functions in terms of PAMP recognition and immune responses. Based on subcellular localization, TLRs are of two types: cell surface types (TLR1, 2, 4, 5, 6, 10, and 11) and endosomal types (TLR3, 7, 8, 9, 12, and 13) [18]. TLR2 heterodimerizes with TLR1 or TLR6, and they share an m-shaped structure. The TLR2-TLR1 heterodimer recognizes triacyl lipopeptides from Gram-negative bacteria and mycoplasma, whereas the TLR2-TLR6 heterodimer recognizes diacyl lipopeptides from Gram-positive bacteria and mycoplasma. For example, in the TLR2-TLR1 heterodimer, TLR2 interacts with two of the three lipid chains of Pam3CSK4 (a triacylated lipopeptide) and the third chain binds the hydrophobic channel of TLR1 [1, 17, 21, 22]. TLR3 was previously reported to recognize double-stranded RNA (dsRNA) produced by numerous viruses during replication or a synthetic analog of dsRNA, polyinosinic-polycytidylic acid (poly(I:C)), which mimics viral infection and induces antiviral immune responses by inducing type I interferons (IFNs) and inflammatory cytokines through the interaction of its ectodomain with dsRNA [23–25]. TLR4 was identified as the long-sought receptor that responds to bacterial lipopolysaccharide (LPS), a component of the outer membrane of Gram-negative bacteria that can cause septic shock [18, 26]. TLR4 heterodimerizes with cell surface MD2, and the complex serves as an LPS-binding component [27]. TLR5 recognizes the flagellin in bacterial flagella [18]. TLR7 reportedly recognizes imidazoquinoline derivatives, guanine analogs including loxoribine; ssRNA derived from RNA viruses such as vesicular stomatitis virus, influenza A virus, and HIV; and certain siRNAs [18, 28, 29]. Mouse TLR8 shares the highest homology with TLR7; however, it is potentially nonfunctional, and human TLR8 recognizes imidazoquinolines and ssRNA. TLR8 is upregulated in monocytes upon bacterial infection [1, 30–32]. TLR9 recognizes unmethylated 2′-deoxyribo CpG DNA motifs in bacteria and viruses, and the sugar backbone of DNA is important for TLR9 recognition [33–35]. TLR9 directly recognizes the insoluble crystal hemozoin, which is generated as a byproduct of the detoxification process after digestion of host hemoglobin by *Plasmodium falciparum* [36, 37]. PAMP recognition by TLRs triggers intracellular signaling pathways to produce inflammatory cytokines, type I IFNs, and chemokines for innate immune responses (Figure 1).

## 3. Cellular Functions of TLRs in NK Cells

### 3.1. Expression of TLRs on NK Cells

NK cells were previously (in the 1970s) reported as large granular circulating lymphocytes accounting for approximately 10–15% of the total blood cells and exhibiting “natural cytotoxicity” against tumor cells by releasing perforin- and granzyme-containing cytotoxic granules [38–40]. Furthermore, they protect the host by limiting viral and bacterial infections before the initiation of the adaptive immune response via activating macrophages, DCs, and neutrophils [12, 38]. Although the expression and cellular functions of TLRs have been extensively studied in macrophages, numerous recent studies have reported that TLRs are the first-line defense in NK cells via TLR-mediated signaling pathways against bacterial, viral, and fungal pathogens [38, 41–43]. Different TLRs are expressed in NK cells, and TLR ligands can activate NK cells directly or indirectly. In human NK cells, *TLR1*–*TLR9* mRNA was reportedly expressed, *TLR1* mRNA levels peaking, followed by *TLR2*, *TLR3*, *TLR5*, and *TLR6* mRNA at moderate levels, while *TLR9* mRNA expression levels were low or undetectable [6, 44, 45].

### 3.2. TLR-Induced Cellular Signaling Pathways

The presence of TLRs has been directly demonstrated through the activation of purified NK cells by TLR ligands and agonists. TLRs are expressed on NK cells independently and can cooperate with chemokines or cytokines to activate NK cell functions including cytokine production and cytotoxicity [11, 45]. As shown in Figure 1, TLRs are activated through specific PAMPs and then differentially induce signaling pathways in NK cells. After ligand or agonist binding to TLRs, TLRs dimerize and undergo conformational changes to recruit downstream adaptor proteins [13] including myeloid differentiation primary response gene 88 (MyD88), TIR domain-containing adaptor protein (TIRAP)/MyD88-adaptor-like (Mal), TIR domain-containing adaptor inducing IFN-*β* (TRIF)/TIR domain-containing adaptor molecule-1 (TICAM-1), and TRIF-related adaptor molecule (TRAM). MyD88 mediates intracellular signaling downstream of all TLRs except for TLR3 [38] (Figure 1). Interaction of adaptor proteins with TLRs is influenced by both the coligation of TLRs with their ligands and oligomerization of TLRs. TLRs activate nuclear factor *κ*B- (NF-*κ*B-) dependent and NF-*κ*B-independent pathways to generate cytokines and chemokines [38]. Interaction of MyD88 with IL-1R-associated kinases (IRAKs) activates a complex containing TNF receptor-associated factor 6 (TRAF6) and TAB2, thus activating TGF*β*-activated kinase 1 (TAK1). TAK1 is critical to determine the differential pathways to activate the NF-*κ*B signaling pathway and mitogen-activated protein kinase pathways [45–47]. Briefly, MyD88 contains an N-terminal death domain (DD), which is separated from its C-terminal TIR domain by a short linker sequence [13, 48–50]. TIRAP is a second TIR-domain-containing adapter. Unlike MyD88, TIRAP does not contain a DD [51, 52]. TRIF was a third TIR-domain-containing adaptor and was identified as a TLR3-binding molecule, also referred to as TICAMI [53, 54]. TRAM is a fourth TIR-domain-containing adaptor identified on the basis of sequence homology in database searches [55]. TRAM interacts with TRIF and TLR4 but not TLR3 [13, 56].

**Figure 2:**
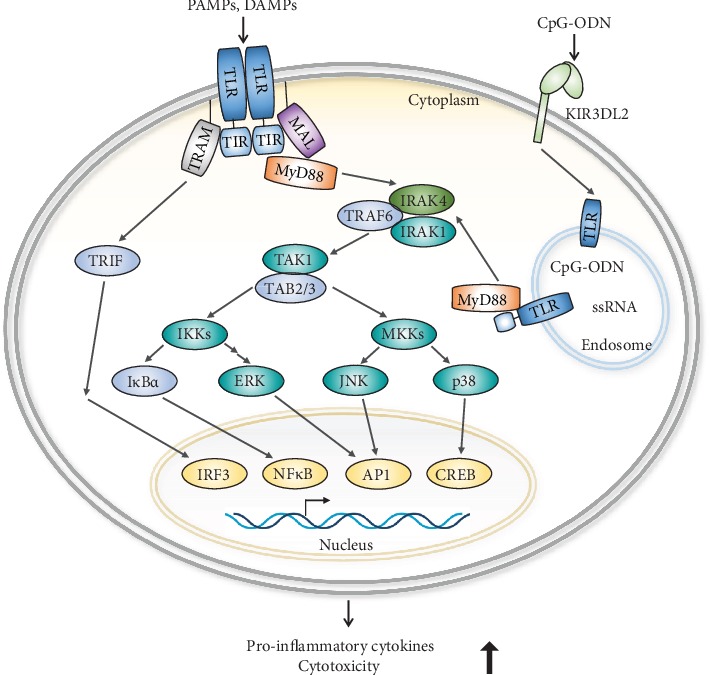
Overview of TLR-mediated signaling pathways. Activated TLRs trigger the association of adaptor proteins and activate their downstream molecules to induce the production of cytokines and cytotoxicity of NK cells.

The IRAK family comprises IRAK1, 2, 3, and 4 and IRAK-M. IRAKs contain an N-terminal DD and a central serine/threonine-kinase domain. IRAK1 and 4 exert kinase activity, whereas IRAK2 and IRAK-M have no detectable kinase activity [57]. TRAF6 comprises six members of the TRAF family in mammals, and they comprise an N-terminal coiled-coil domain and a conserved C-terminal domain. TAK1 and TAB1/2 regulate TRAF6-induced activation of NF-*κ*B and activator protein 1 (AP1) transcription factor. Finally, transcription factors are activated to transcribe their target cytokines, chemokines, and mediators of immune responses (Figure 2).

In addition, TLR ligands or agonists differentially regulate TLR-mediated signaling pathways in NK cells. Numerous studies have demonstrated stimulation of TLRs by TLR ligands or agonists and reported the differential activation of NK cells by them. *K. pneumoniae* OmpA and flagellin reportedly stimulated TLR2 and 5 and induced IFN-*γ* and *α*-defensin production in human NK cells [44]. *M. bovis* and *H. pylori* (HpaA lipoprotein) stimulated TLR2 and induced CD69 and CD25 expression and IFN-*γ* and TNF production and IFN-*γ* production, respectively, in human NK cells [58, 59]. Moreover, diacyl lipopeptide reportedly induced IFN-*γ* production and cytotoxicity in mouse NK cells via TLR2 stimulation [60]. Poly (I:C) stimulated TLR3 to induce cytotoxicity and CXCL10 and IFN-*γ* production in human NK cells [61]. Another study reported that Poly (I:C) and loxoribine stimulate TLR3 and 7 and induce IFN-*γ* production and cytotoxicity in human NK cells [62]. Poly (I:C) and CpG stimulated TLR3 and 9 in human NK cells and upregulated CD69 and CD25 and increased cytotoxicity and IFN-*γ* and TNF production [63]. Peptidoglycan, Poly (I:C), LPS, and flagellin stimulated TLR2, 3, 4, and 5 and induced cytotoxicity and IFN-*γ* production in human NK cells [45]. Peptidoglycan and Poly (I:C) stimulated TLR2, 3, and 7 to induce IFN-*γ* production and cytotoxicity in mouse NK cells [64]. The CpG oligonucleotide reportedly serves as a TLR9 agonist and induces CD69 expression, thus suppressing bacterial growth in human and mouse NK cells, respectively [65, 66]. Although TLR agonists can directly activate NK cells, the microenvironment plays a potential role in activating their cytotoxicity and regulatory functions during TLR-mediated activation to induce subsequent immune responses [1, 67, 68].

## 4. Application of TLR Agonists for NK Cell-Mediated Therapy

TLR-mediated signaling pathways efficiently activate the effector functions of NK cells *in vitro* and *in vivo*. A number of clinical trials investigated the immunotherapeutic anticancer property of NK cells in various patient populations [69]. Interestingly, TLR agonists are potentially applicable to enhance the therapeutic effector function of NK cells for caner immunotherapy.

Trastuzumab is a humanized anti-HER2 monoclonal antibody (mAb) and is the first HER2-targeted therapy approved by the Food and Drug Administration. Trastuzumab has significantly advanced the clinical management of patients with HER2^+^ breast cancer by prolonging disease-free survival and overall survival in patients with early-stage breast cancer, and progression-free survival and overall survival in patients with metastatic breast cancer [70, 71]. The therapeutic effect of trastuzumab therapy is partially dependent on functional NK cells. NK cell recognition of antibody-coated tumor cells through surface Fc*γ*RIII/CD16 provides a potent activation signal leading to antibody-dependent cell-mediated cytotoxicity (ADCC) [72, 73]. A polysaccharide krestin (PSK), a natural product extracted from medicinal mushroom *Trametes versicolor*, has recently been considered a potent TLR2 agonist. The effect of PSK on human NK cells and the potential of PSK to enhance HER2-targeted mAb therapy has been investigated. PSK activates human NK cells to produce IFN-*γ* and to lyse K562 target cells, enhances trastuzumab-mediated ADCC against SKBR3 and MDA-MB-231 breast cancer cells, and activates human NK cells and potentiates trastuzumab-mediated ADCC. Concurrently, PSK and trastuzumab therapy is a potentially novel method to induce the antitumor effect of trastuzumab [74].

TLR3 is an endosomal receptor that senses viral dsRNA [75]. Sensing of viral dsRNA by TLR3 leads to the secretion of type I IFN and other proinflammatory cytokines [23]. The TLR3 agonist Poly (I:C) reportedly suppressed tumor growth in mice [76, 77], and TLR3 agonists have been assessed in phase I/II trials as adjuvants for therapeutic vaccination against melanoma and breast cancer [78]. TLR3 reportedly limited experimental B16F10 lung metastasis, an immunologic constraint dependent on both IFN-*γ* secretion and NK cells, and NK cells derived from Tlr3 null mice were hyporesponsive to cytokine stimulation, thus suggesting a pivotal role of endogenous TLR3 stimulation in the acquisition of complete NK cell functions and immune protection against experimental metastasis [79].

Synthetic TLR7 ligands induced a type 1 interferon response along with the secretion of proinflammatory cytokines including IL-1b, IL-6, and IL-12 by recruiting MyD88, interferon regulatory factors, and NF-*κ*B [80–82]. A novel small-molecule agonist, SC1, has been developed for TLR7, and *in vivo* studies have attempted to determine the mode of action of SC1. Mice bearing the NK cell-sensitive lymphoma RMA-S were cured via repeated s.c. SC1 administration. SC1 treatment reportedly activated NK cells in a TLR7- and IFN-*α*-dependent manner, and SC1 thus reverses NK cell anergy leading to efficient tumor cell lysis [83].

The anti-CD20 monoclonal antibody (mAb) rituximab reportedly significantly improved patient survival; however, numerous patients ultimately experience relapse, thus necessitating the development of novel therapies and improved anti-CD20 mAbs [84, 85]. Immune stimulation through TLR7 activation in combination with obinutuzumab is hypothesized to further enhance lymphoma clearance and the generation of long-term antitumor immune responses. In syngeneic human CD20- (hCD20-) expressing models of lymphoma, systemic administration of a TLR7 agonist (R848) reportedly augmented responses upon combinatorial administration with obinutuzumab, thus preventing tumor recurrence. Furthermore, primary antitumor activity depended on both NK cells and CD4^+^ T cells but not on CD8^+^ T cells, suggesting that combinatorial treatment with TLR7 agonists potentially improves the outcome of obinutuzumab treatment [86].

ADCC is a well-established effector pathway that contributes to the mAb-mediated therapies including cetuximab, an epidermal growth factor receptor- (EGFR-) specific mAb approved for treating squamous cell carcinoma of the head and neck (SCCHN). VTX-2337 is a selective TLR8 agonist that is more potent than either resiquimod (R848) or 3M-002 (CL075), which is currently in phase II clinical trials for multiple oncological indications [87]. Cetuximab, a clinically approved, epidermal growth factor receptor-specific monoclonal antibody, activates NK cells through interactions with Fc*γ*RIII and facilitates ADCC in tumor cells. A phase I open-label, dose escalation trial including 13 patients with recurrent or metastatic SCCHN reported that patient NK cells become more responsive to stimulation by NKG2D or Fc*γ*RIII after VTX-2337 treatment, suggesting that TLR8 stimulation and inflammasome activation by VTX-2337 potentially complements Fc*γ*RIII engagement and augments clinical responses in SCCHN patients treated with cetuximab [88].

NK cells play an important role in the host response against various pathogens. NK cells can detect and damage various viral, bacterial, and fungal pathogens and also modulate or activate a variety of cells in the innate and adaptive immune system. NK cells are active against pathogens, and animal studies suggested that NK cells could be applied in the antimicrobial immunotherapy [69].

Over the past decade, the effect of NK cells in controlling HIV-1 infections *in vivo* has been reported [89, 90]. TLR agonists are potent enhancers of innate antiviral immunity and potentially reverse HIV-1 latency. Studies have attempted to improve NK cell function, using TLR9 agonists, suggesting that a novel TLR9 agonist, MGN1703, is potentially effective in an HIV-1 eradication trial [91]. Incubation of peripheral blood mononuclear cells with MGN1703 reportedly resulted in NK cell activation and increased NK cell function, thus significantly inhibiting the spread of HIV in a culture of autologous CD4^+^ T cells. MGN1703 induced strong antiviral innate immune responses, enhanced HIV-1 transcription, and boosted NK cell-mediated suppression of HIV-1 infections in autologous CD4^+^ T cells, suggesting that the preclinical basis for an HIV eradication clinical trial is the inclusion of MGN1703 [92].

**Figure 3:**
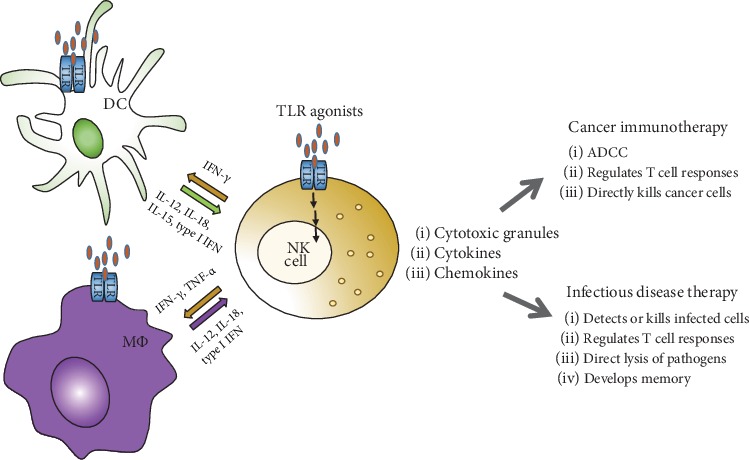
Application of TLR agonists for NK cell-mediated therapy. NK cells are activated directly by TLR agonists through TLRs or indirectly by NK cell-activating cytokines released by dendritic cells (DCs) and macrophages (M*Φ*). NK cells also activate DCs and macrophages by secretion of IFN-*γ* and TNF-*α*.

NK cell activation during TLR stimulation by TLR agonists including bacteria-associated peptidoglycan, LPS, virus-derived dsRNA, and DNA with CpG motifs can be potently and indirectly induced by cytokines released by coexisting dendritic cells (DCs) and macrophages at sites of infection [93–95]. The activation of NK cells by DCs is dependent on both cell-to-cell interaction and soluble factors [96, 97]. DC-derived IL-12, IL-15, IL-18, and type I IFN are crucial for the production of IFN-*γ* in NK cells, and NK cell-derived IFN-*γ* then facilitates the activation of DCs. They have a positive feedback loop that amplifies TLR-induced activation of NK cells and DCs [95, 98–100]. Macrophages secrete IL-12, IL-18, and type I IFN to activate NK cells during microbial infection through TLR signaling pathways. Activated NK cells induce antimicrobial functions of macrophages by producing IFN-*γ* and TNF-*α* [94, 95]. Although these positive feedback loops between NK cells and DCs or macrophages facilitate beneficial functions of microbial clearance, the excessive production of cytokines can induce systemic inflammation *in vivo* [95] (Figure 3).

## 5. Conclusion

NK cells play an important role in the host response against various pathogens. TLRs are expressed on innate immune cells or some adaptive immune cells and mediate innate immune responses against microbial pathogens and induce adaptive immune responses. TLRs are also expressed in NK cells, and TLR ligands can activate NK cells directly or indirectly. Recent studies have reported that TLRs perform the first-line defense in NK cells against bacterial and viral infections by inducing NK cytotoxicity and cytokine production. TLR agonists were suggested as potential boosters in stimulating immunological effector function of NK cells for cancer immunotherapy and infectious disease therapy. However, to develop new drugs targeting TLRs, we should understand the complex mechanisms underlying TLR localization and function in NK cells. It will provide data for novel therapeutic tools involving TLRs and their agonists, and these approaches may be promising and have an important clinical impact for immunotherapy using NK cells in the future.

## References

[1] Kawai T., Akira S. (2010). The role of pattern-recognition receptors in innate immunity: update on Toll-like receptors. *Nature Immunology*.

[2] Takeda K., Akira S. (2005). Toll-like receptors in innate immunity. *International Immunology*.

[3] Yoneyama M., Fujita T. (2009). RNA recognition and signal transduction by RIG-I-like receptors. *Immunological Reviews*.

[4] Lee C. C., Avalos A. M., Ploegh H. L. (2012). Accessory molecules for Toll-like receptors and their function. *Nature Reviews Immunology*.

[5] O'Neill L. A., Golenbock D., Bowie A. G. (2013). The history of Toll-like receptors - redefining innate immunity. *Nature Reviews Immunology*.

[6] Adib-Conquy M., Scott-Algara D., Cavaillon J. M., Souza-Fonseca-Guimaraes F. (2014). TLR-mediated activation of NK cells and their role in bacterial/viral immune responses in mammals. *Immunology and Cell Biology*.

[7] Nowarski R., Gagliani N., Huber S., Flavell R. A. (2013). Innate immune cells in inflammation and cancer. *Cancer Immunology Research*.

[8] Souza-Fonseca-Guimaraes F., Adib-Conquy M., Cavaillon J. M. (2012). Natural killer (NK) cells in antibacterial innate immunity: angels or devils?. *Molecular Medicine*.

[9] Hargreaves D. C., Medzhitov R. (2005). Innate sensors of microbial infection. *Journal of Clinical Immunology*.

[10] Zitti B., Bryceson Y. T. (2018). Natural killer cells in inflammation and autoimmunity. *Cytokine & Growth Factor Reviews*.

[11] Sivori S., Carlomagno S., Pesce S., Moretta A., Vitale M., Marcenaro E. (2014). TLR/NCR/KIR: which one to use and when?. *Frontiers in Immunology*.

[12] Vivier E., Raulet D. H., Moretta A. (2011). Innate or adaptive immunity? The example of natural killer cells. *Science*.

[13] Akira S., Takeda K. (2004). Toll-like receptor signalling. *Nature Reviews Immunology*.

[14] Beutler B. (2004). Inferences, questions and possibilities in Toll-like receptor signalling. *Nature*.

[15] Wagner H. (2006). Endogenous TLR ligands and autoimmunity. *Advances in Immunology*.

[16] Vaknin I., Blinder L., Wang L. (2008). A common pathway mediated through Toll-like receptors leads to T- and natural killer-cell immunosuppression. *Blood*.

[17] Kawai T., Akira S. (2006). TLR signaling. *Cell Death and Differentiation*.

[18] Akira S., Uematsu S., Takeuchi O. (2006). Pathogen recognition and innate immunity. *Cell*.

[19] Xu Y., Tao X., Shen B. (2000). Structural basis for signal transduction by the Toll/interleukin-1 receptor domains. *Nature*.

[20] Patel H., Shaw S. G., Shi-Wen X., Abraham D., Baker D. M., Tsui J. C. (2012). Toll-like receptors in ischaemia and its potential role in the pathophysiology of muscle damage in critical limb ischaemia. *Cardiology Research and Practice*.

[21] Jin M. S., Kim S. E., Heo J. Y. (2007). Crystal structure of the TLR1-TLR2 heterodimer induced by binding of a tri-acylated lipopeptide. *Cell*.

[22] Kang J. Y., Nan X., Jin M. S. (2009). Recognition of lipopeptide patterns by Toll-like receptor 2-Toll-like receptor 6 heterodimer. *Immunity*.

[23] Alexopoulou L., Holt A. C., Medzhitov R., Flavell R. A. (2001). Recognition of double-stranded RNA and activation of NF-kappaB by Toll-like receptor 3. *Nature*.

[24] Choe J., Kelker M. S., Wilson I. A. (2005). Crystal structure of human toll-like receptor 3 (TLR3) ectodomain. *Science*.

[25] Bell J. K., Askins J., Hall P. R., Davies D. R., Segal D. M. (2006). The dsRNA binding site of human Toll-like receptor 3. *Proceedings of the National Academy of Sciences of the United States of America*.

[26] Hoshino K., Takeuchi O., Kawai T. (1999). Cutting edge: Toll-like receptor 4 (TLR4)-deficient mice are hyporesponsive to lipopolysaccharide: evidence for TLR4 as the Lps gene product. *Journal of Immunology*.

[27] Akashi-Takamura S., Miyake K. (2008). TLR accessory molecules. *Current Opinion in Immunology*.

[28] Kawai T., Akira S. (2006). Innate immune recognition of viral infection. *Nature Immunology*.

[29] Hornung V., Guenthner-Biller M., Bourquin C. (2005). Sequence-specific potent induction of IFN-alpha by short interfering RNA in plasmacytoid dendritic cells through TLR7. *Nature Medicine*.

[30] Heil F., Hemmi H., Hochrein H. (2004). Species-specific recognition of single-stranded RNA via toll-like receptor 7 and 8. *Science*.

[31] Heil F., Ahmad-Nejad P., Hemmi H. (2003). The Toll-like receptor 7 (TLR7)-specific stimulus loxoribine uncovers a strong relationship within the TLR7, 8 and 9 subfamily. *European Journal of Immunology*.

[32] Jurk M., Heil F., Vollmer J. (2002). Human TLR7 or TLR8 independently confer responsiveness to the antiviral compound R-848. *Nature Immunology*.

[33] Hemmi H., Takeuchi O., Kawai T. (2000). A Toll-like receptor recognizes bacterial DNA. *Nature*.

[34] Krug A., French A. R., Barchet W. (2004). TLR9-dependent recognition of MCMV by IPC and DC generates coordinated cytokine responses that activate antiviral NK cell function. *Immunity*.

[35] Lund J., Sato A., Akira S., Medzhitov R., Iwasaki A. (2003). Toll-like receptor 9-mediated recognition of herpes simplex virus-2 by plasmacytoid dendritic cells. *The Journal of Experimental Medicine*.

[36] Coban C., Ishii K. J., Kawai T. (2005). Toll-like receptor 9 mediates innate immune activation by the malaria pigment hemozoin. *The Journal of Experimental Medicine*.

[37] Coban C., Igari Y., Yagi M. (2010). Immunogenicity of whole-parasite vaccines against plasmodium falciparum involves malarial hemozoin and host TLR9. *Cell Host & Microbe*.

[38] Yang Z., Kong B., Mosser D. M., Zhang X. (2011). TLRs, macrophages, and NK cells: our understandings of their functions in uterus and ovary. *International Immunopharmacology*.

[39] Kiessling R., Klein E., Wigzell H. (1975). "Natural" killer cells in the mouse. I. Cytotoxic cells with specificity for mouse Moloney leukemia cells. Specificity and distribution according to genotype. *European Journal of Immunology*.

[40] Herberman R. B., Nunn M. E., Holden H. T., Lavrin D. H. (1975). Natural cytotoxic reactivity of mouse lymphoid cells against syngeneic and allogeneic tumors. II. Characterization of effector cells. *International journal of cancer*.

[41] Cerwenka A., Lanier L. L. (2001). Natural killer cells, viruses and cancer. *Nature Reviews Immunology*.

[42] Della Chiesa M., Marcenaro E., Sivori S., Carlomagno S., Pesce S., Moretta A. (2014). Human NK cell response to pathogens. *Seminars in Immunology*.

[43] Bar E., Whitney P. G., Moor K., e Sousa C. R., LeibundGut-Landmann S. (2014). IL-17 regulates systemic fungal immunity by controlling the functional competence of NK cells. *Immunity*.

[44] Chalifour A., Jeannin P., Gauchat J. F. (2004). Direct bacterial protein PAMP recognition by human NK cells involves TLRs and triggers alpha-defensin production. *Blood*.

[45] Lauzon N. M., Mian F., MacKenzie R., Ashkar A. A. (2006). The direct effects of Toll-like receptor ligands on human NK cell cytokine production and cytotoxicity. *Cellular Immunology*.

[46] Takeda K., Kaisho T., Akira S. (2003). Toll-like receptors. *Annual Review of Immunology*.

[47] Dunne A., O'Neill L. A. (2003). The interleukin-1 receptor/Toll-like receptor superfamily: signal transduction during inflammation and host defense. *Science Signaling*.

[48] Muzio M., Ni J., Feng P., Dixit V. M. (1997). IRAK (Pelle) family member IRAK-2 and MyD88 as proximal mediators of IL-1 signaling. *Science*.

[49] Wesche H., Henzel W. J., Shillinglaw W., Li S., Cao Z. (1997). MyD88: an adapter that recruits IRAK to the IL-1 receptor complex. *Immunity*.

[50] Burns K., Martinon F., Esslinger C. (1998). MyD88, an adapter protein involved in interleukin-1 signaling. *The Journal of Biological Chemistry*.

[51] Horng T., Barton G. M., Medzhitov R. (2001). TIRAP: an adapter molecule in the Toll signaling pathway. *Nature Immunology*.

[52] Fitzgerald K. A., Palsson-McDermott E. M., Bowie A. G. (2001). Mal (MyD88-adapter-like) is required for Toll-like receptor-4 signal transduction. *Nature*.

[53] Yamamoto M., Sato S., Mori K. (2002). Cutting edge: a novel Toll/IL-1 receptor domain-containing adapter that preferentially activates the IFN-*β* promoter in the Toll-like receptor signaling. *Journal of Immunology*.

[54] Oshiumi H., Matsumoto M., Funami K., Akazawa T., Seya T. (2003). TICAM-1, an adaptor molecule that participates in Toll-like receptor 3-mediated interferon-beta induction. *Nature Immunology*.

[55] Yamamoto M., Sato S., Hemmi H. (2003). TRAM is specifically involved in the Toll-like receptor 4-mediated MyD88-independent signaling pathway. *Nature Immunology*.

[56] Fitzgerald K. A., Rowe D. C., Barnes B. J. (2003). LPS-TLR4 signaling to IRF-3/7 and NF-kappaB involves the toll adapters TRAM and TRIF. *The Journal of Experimental Medicine*.

[57] Janssens S., Beyaert R. (2003). Functional diversity and regulation of different interleukin-1 receptor-associated kinase (IRAK) family members. *Molecular Cell*.

[58] Marcenaro E., Ferranti B., Falco M., Moretta L., Moretta A. (2008). Human NK cells directly recognize Mycobacterium bovis via TLR2 and acquire the ability to kill monocyte-derived DC. *International Immunology*.

[59] Lindgren A., Pavlovic V., Flach C. F., Sjoling A., Lundin S. (2011). Interferon-gamma secretion is induced in IL-12 stimulated human NK cells by recognition of helicobacter pylori or TLR2 ligands. *Innate Immunity*.

[60] Azuma M., Sawahata R., Akao Y. (2010). The peptide sequence of diacyl lipopeptides determines dendritic cell TLR2-mediated NK activation. *PLoS One*.

[61] Pisegna S., Pirozzi G., Piccoli M., Frati L., Santoni A., Palmieri G. (2004). p38 MAPK activation controls the TLR3-mediated up-regulation of cytotoxicity and cytokine production in human NK cells. *Blood*.

[62] Girart M. V., Fuertes M. B., Domaica C. I., Rossi L. E., Zwirner N. W. (2007). Engagement of TLR3, TLR7, and NKG2D regulate IFN-gamma secretion but not NKG2D-mediated cytotoxicity by human NK cells stimulated with suboptimal doses of IL-12. *Journal of Immunology*.

[63] Sivori S., Falco M., Della Chiesa M. (2004). CpG and double-stranded RNA trigger human NK cells by Toll-like receptors: induction of cytokine release and cytotoxicity against tumors and dendritic cells. *Proceedings of the National Academy of Sciences*.

[64] Sawaki J., Tsutsui H., Hayashi N. (2007). Type 1 cytokine/chemokine production by mouse NK cells following activation of their TLR/MyD88-mediated pathways. *International Immunology*.

[65] Hornung V., Rothenfusser S., Britsch S. (2002). Quantitative expression of toll-like receptor 1-10 mRNA in cellular subsets of human peripheral blood mononuclear cells and sensitivity to CpG oligodeoxynucleotides. *Journal of Immunology*.

[66] Elkins K. L., Colombini S. M., Krieg A. M., De Pascalis R. (2009). NK cells activated in vivo by bacterial DNA control the intracellular growth of Francisella tularensis LVS. *Microbes and Infection*.

[67] Marcenaro E., Carlomagno S., Pesce S., Moretta A., Sivori S. (2011). Bridging innate NK cell functions with adaptive immunity. *Advances in Experimental Medicine and Biology*.

[68] Marcenaro E., Della Chiesa M., Bellora F. (2005). IL-12 or IL-4 prime human NK cells to mediate functionally divergent interactions with dendritic cells or tumors. *Journal of Immunology*.

[69] Schmidt S., Tramsen L., Rais B., Ullrich E., Lehrnbecher T. (2018). Natural killer cells as a therapeutic tool for infectious diseases-current status and future perspectives. *Oncotarget*.

[70] Hudis C. A. (2007). Trastuzumab--mechanism of action and use in clinical practice. *The New England Journal of Medicine*.

[71] Baselga J., Perez E. A., Pienkowski T., Bell R. (2006). Adjuvant trastuzumab: a milestone in the treatment of HER-2-positive early breast cancer. *The oncologist*.

[72] Diefenbach A., Raulet D. H. (2002). The innate immune response to tumors and its role in the induction of T-cell immunity. *Immunological Reviews*.

[73] Kaifu T., Escaliere B., Gastinel L. N., Vivier E., Baratin M. (2011). B7-H6/NKp30 interaction: a mechanism of alerting NK cells against tumors. *Cellular and molecular life sciences*.

[74] Lu H., Yang Y., Gad E. (2011). TLR2 agonist PSK activates human NK cells and enhances the antitumor effect of HER2-targeted monoclonal antibody therapy. *Clinical cancer research*.

[75] Matsumoto M., Seya T. (2008). TLR3: interferon induction by double-stranded RNA including poly(I:C). *Advanced Drug Delivery Reviews*.

[76] Levy H. B., Law L. W., Rabson A. S. (1969). Inhibition of tumor growth by polyinosinic-polycytidylic acid. *Proceedings of the National Academy of Sciences*.

[77] Forte G., Rega A., Morello S. (2012). Polyinosinic-polycytidylic acid limits tumor outgrowth in a mouse model of metastatic lung cancer. *Journal of Immunology*.

[78] Sharma S., Zhu L., Davoodi M. (2013). TLR3 agonists and proinflammatory antitumor activities. *Expert Opinion on Therapeutic Targets*.

[79] Guillerey C., Chow M. T., Miles K. (2015). Toll-like receptor 3 regulates NK cell responses to cytokines and controls experimental metastasis. *Oncoimmunology*.

[80] Hemmi H., Kaisho T., Takeuchi O. (2002). Small anti-viral compounds activate immune cells via the TLR7 MyD88-dependent signaling pathway. *Nature Immunology*.

[81] Bourquin C., Schmidt L., Lanz A. L. (2009). Immunostimulatory RNA oligonucleotides induce an effective antitumoral NK cell response through the TLR7. *Journal of Immunology*.

[82] Gilliet M., Cao W., Liu Y. J. (2008). Plasmacytoid dendritic cells: sensing nucleic acids in viral infection and autoimmune diseases. *Nature Reviews Immunology*.

[83] Wiedemann G. M., Jacobi S. J., Chaloupka M. (2016). A novel TLR7 agonist reverses NK cell anergy and cures RMA-S lymphoma-bearing mice. *OncoImmunology*.

[84] Coiffier B., Lepage E., Briere J. (2002). CHOP chemotherapy plus rituximab compared with CHOP alone in elderly patients with diffuse large-B-cell lymphoma. *The New England Journal of Medicine*.

[85] Hallek M., Fischer K., Fingerle-Rowson G. (2010). Addition of rituximab to fludarabine and cyclophosphamide in patients with chronic lymphocytic leukaemia: a randomised, open-label, phase 3 trial. *The Lancet*.

[86] Cheadle E. J., Lipowska-Bhalla G., Dovedi S. J. (2017). A TLR7 agonist enhances the antitumor efficacy of obinutuzumab in murine lymphoma models via NK cells and CD4 T cells. *Leukemia*.

[87] Lu H., Dietsch G. N., Matthews M. A. (2012). VTX-2337 is a novel TLR8 agonist that activates NK cells and augments ADCC. *Clinical cancer research*.

[88] Dietsch G. N., Lu H., Yang Y. (2016). Coordinated activation of Toll-like receptor8 (TLR8) and NLRP3 by the TLR8 agonist, VTX-2337, ignites tumoricidal natural killer cell activity. *PloS one*.

[89] Alter G., Heckerman D., Schneidewind A. (2011). HIV-1 adaptation to NK-cell-mediated immune pressure. *Nature*.

[90] Sips M., Sciaranghella G., Diefenbach T. (2012). Altered distribution of mucosal NK cells during HIV infection. *Mucosal Immunology*.

[91] Schmidt M., Hagner N., Marco A., Konig-Merediz S. A., Schroff M., Wittig B. (2015). Design and structural requirements of the potent and safe TLR-9 agonistic immunomodulator MGN1703. *Nucleic Acid Therapeutics*.

[92] Offersen R., Nissen S. K., Rasmussen T. A. (2016). A novel Toll-like receptor 9 agonist, MGN1703, enhances HIV-1 transcription and NK cell-mediated inhibition of HIV-1-infected autologous CD4+ T cells. *Journal of Virology*.

[93] Souza-Fonseca-Guimaraes F., Parlato M., Philippart F., Misset B., Cavaillon J. M., Adib-Conquy M. (2012). Toll-like receptors expression and interferon-*γ* production by NK cells in human sepsis. *Critical care*.

[94] Varma T. K., Lin C. Y., Toliver-Kinsky T. E., Sherwood E. R. (2002). Endotoxin-induced gamma interferon production: contributing cell types and key regulatory factors. *Clinical and Diagnostic Laboratory Immunology*.

[95] Guo Y., Patil N. K., Luan L., Bohannon J. K., Sherwood E. R. (2018). The biology of natural killer cells during sepsis. *Immunology*.

[96] Fernandez N. C., Lozier A., Flament C. (1999). Dendritic cells directly trigger NK cell functions: cross-talk relevant in innate anti-tumor immune responses in vivo. *Nature Medicine*.

[97] Yu Y., Hagihara M., Ando K. (2001). Enhancement of human cord blood CD34+ cell-derived NK cell cytotoxicity by dendritic cells. *Journal of Immunology*.

[98] Ferlazzo G., Pack M., Thomas D. (2004). Distinct roles of IL-12 and IL-15 in human natural killer cell activation by dendritic cells from secondary lymphoid organs. *Proceedings of the National Academy of Sciences*.

[99] Anguille S., Van Acker H. H., Van den Bergh J. (2015). Interleukin-15 dendritic cells harness NK cell cytotoxic effector function in a contact- and IL-15-dependent manner. *PLoS One*.

[100] Semino C., Angelini G., Poggi A., Rubartelli A. (2005). NK/iDC interaction results in IL-18 secretion by DCs at the synaptic cleft followed by NK cell activation and release of the DC maturation factor HMGB1. *Blood*.

